# Supplementary material for: Attenuation of Myocardial Dysfunction in Hypertensive Cardiomyopathy Using Non-R-Wave-Synchronized Cardiac Shock Wave Therapy
Source: Int J Mol Sci. 2022 Oct 31;23(21):13274. doi: 10.3390/ijms232113274 (PMC9654370; doi:10.3390/ijms232113274)
Supplement: Supplementary file 1 [file ijms-23-13274-s001.zip › ijms-1927487-supplementary.pdf]

Supplemental Material For

Attenuation of Myocardial Dysfunction in Hypertensive Cardiomyopathy using Non-R wave Synchronized Cardiac Shock Wave Therapy

This file includes:

Supplemental Figure S1. And it is on page 2.

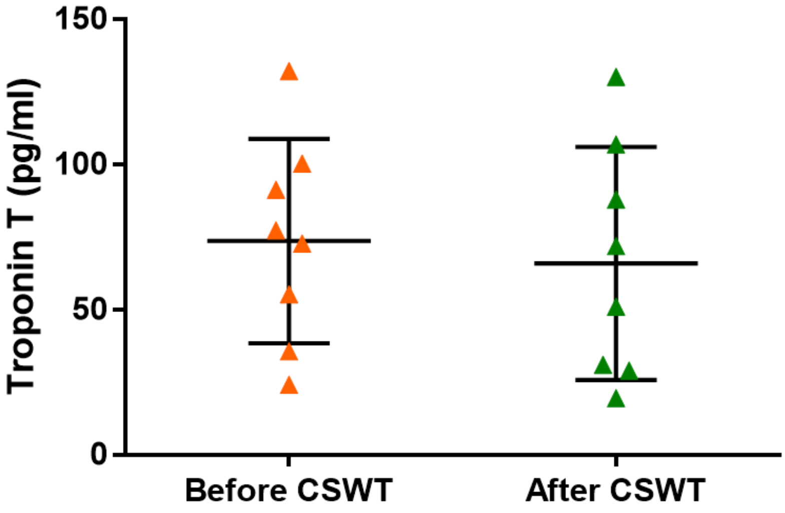

Supplemental Figure S1. Troponin T level after CSWT. No significant plasma Troponin T level was observed before and after CSWT.
